# Evaluation of antibacterial activity of honey against multidrug resistant bacteria in Ayder Referral and Teaching Hospital, Northern Ethiopia

**DOI:** 10.1186/s40064-016-2493-x

**Published:** 2016-06-23

**Authors:** Araya Gebereyesus Wasihun, Berhe Gebreslassie Kasa

**Affiliations:** Department of Medical Microbiology and Immunology, Institute of Biomedical Sciences, College of Health Sciences, Mekelle University, Mekelle, Ethiopia; Department of Surgery, School of Medicine, College of Health Sciences, Mekelle University, Mekelle, Ethiopia

**Keywords:** Honey, MIC, MBC, Control bacteria, Test bacteria

## Abstract

**Background:**

Multidrug resistance is a global health issue. Hence integration of traditional medicine like honey and modern medicine could be a best option in the treatment of patients infected with drug resistant bacteria. Despite the multi floral and huge honey production in the region, there are no studies that evaluate the antibacterial activity of honey against multidrug resistant bacteria.

**Objective:**

To evaluate the antibacterial activity of honey against multidrug resistant human pathogenic bacterial isolates of wound and ear infections.

**Methods:**

Red and white honeys were obtained from three districts Eastern Zone of Tigray namely Temben, Atsbi and Samre. The antibacterial potential of these honeys was determined against multidrug resistant isolates of clinical isolates of bacterial species of *Staphylococcus aureus*, *Escherichia coli*, *Pseudomonas aeruginosa*, *Proteus mirabilis*, Coagulase negative Staphylococcus, *Streptococcus pyogenes* and *Klebsiella pneumonia*, and five controls bacterial using tube dilutions methods. Undiluted and twofold serial dilutions of honeys were tested to determine minimum inhibitory concentration (MIC) using broth tube dilution methods through visual inspection and minimum bactericidal concentration (MBC) was determined by sub-culturing tubes showing no visible sign of growth/turbidity in MIC.

**Results:**

The mean MIC of red honeys for control and test bacteria was 7.7–8.9 and 12.6–17.9 % (v/v) respectively. Whilst the MIC of white honey was 12.2–12.5 % (v/v) for control and 16.1–27.7 % (v/v) for test bacteria. Mean MBC of red honeys for control and test isolates was from 25–40 to 30.4–62.5 % (v/v) respectively, and 40–55 and 60.7–75 % (v/v) for white honeys. Honey collected from Samre area has shown better antibacterial activity than other sites. Similarly red honeys from all areas were found to have better antibacterial activity against the multidrug bacteria than the white honey. Over all the MIC and MBC of all isolates was between 6.25–50 and 12.5–100 % (v/v) respectively.

**Conclusion:**

Red honey from all sites showed better antibacterial activity than the white honey. Likewise, honey from Samre area showed better antibacterial activity than Temben and Atsbi districts. All collected honeys showed varied bacteriostatic and bactericidal activities, and none of the isolates was resistant to tested honeys.

## Background

The use of traditional and herbal medicine to treat infections was practiced since the origin of mankind, and it was the only option to treat before the ear of antibiotics (Jawad 2011). A variety of plants and their extracts have been used for the treatment requiring antimicrobial activity, and one of the popular natural antimicrobial substances described in the ancient medicine is honey (Mandal and Mandal 2011).

Honey is the natural sweet substance obtained from the secretions of the living parts or excretions of plants which the honey bees (*Apis mellifera*) collect and store (Moore et al. 2001). Though honey is used widely in traditional medicine, its use in modern medicine is limited (Geenwood 1993). Honey is used for the treatment of many infections, and also used effectively as wound dressing including surgical wounds, burns and skin ulcers. Mainly because it speeds up the growth of new tissues and help to heal the wound, reduces pain and odor quickly (Lusby et al. 2002).

The high osmotic nature and naturally low pH (3.2–4.5) (Kwakman and Zaat 2012), ability to produce hydrogen peroxide, which plays a key role in the antimicrobial activity of honey (Kacaniova et al. 2011) and phytochemical factors such as tetracycline derivatives, peroxides, amylase, fatty acids, phenols, ascorbic acid, terpenes, benzyl alcohol and benzoic acid (Bogdanov 1984; Shears 2000) are factors attributed honey to have potent bactericidal and bacteriostatic activity against pathogenic bacteria.

Studies have shown the broad-spectrum antibacterial effect of honey for several bacteria including, aerobes and anaerobes and gram-positive and gram negative (Allen et al. 2000; Kingsley 2001; Al-Waili et al. 2005; Cooper et al. 2002). Most human pathogenic bacteria causing wound infections such as *Staphylococcus aureus*, *Pseudomonas aeruginosa*, *Escherichia coli* and *Streptococcus pyogenes* were found to be sensitive to honey (Visavadia et al. 2006). Concentration of honey used and the nature of the bacterial isolate (Kacaniova et al. 2011); origin and method of honey processing (Molan 1992) are factors that affect the antibacterial activity of honey. This variation in the antibacterial potency of different types of honeys hampered its acceptance in modern medicine (Kwakman 2008).

Over and indiscriminate use of antibiotics has led to the emergence of multidrug resistant bacterial strains, a global public health problem (Kacaniova et al. 2011; Mandal et al. 2009). To solve this challenge, alternative antimicrobial strategies like plants and plant-based products such as honey have currently got more attention (Basualdo et al. 2007; Mulu et al. 2004).

There are few researches conducted in Ethiopia regarding the antimicrobial potential of honey (Ahmed et al. 2014; Getaneh et al. 2013; Mulu et al. 2004; Mogessie 1994). However, research papers explaining both the antimicrobial potential on multidrug resistant bacteria and variations in the antimicrobial potential of different honeys are limited. This study, therefore, not only highlights the antibacterial potential of the honeys but also gives information about the variations in the antibacterial potential of different honeys by color and districts in Eastern Zone of Tigray Regional state against multidrug resistant bacteria isolates.

## Methods

### Study design, study area and period

Experimental study design method was conducted in Mekelle University, College of Health Sciences, Ayder Referral and Teaching Hospital from January to May 2015.

### Specimen collection

Swabs from ear discharge and post surgical wounds were collected from patients attended the ear, nose and throat (ENT) clinic and surgical wards. Specimens were then transported to the microbiology laboratory for bacteriological analysis and experimental test.

### Isolation and identification of bacteria

Collected swabs were inoculated on MacConkey agar, Blood agar and Manitol Salt agar and incubated at 37 °C for 18–24 h aerobically. Grown isolates were then identified by their colony morphology, Gram staining reaction, and Biochemical tests including Catalase test, Coagulase test, Triple Sugar Iron agar (TSI) (OXOID, UK), Citrate utilization test (BBL™), Urease test (BBL™), Motility Indole Lysine (MIL) [BBL™] and Optochin test using the standard procedure for bacterial identification (Clinical and Laboratory Standards Institute 2013). Accordingly the following bacteria were identified from the clinical specimens isolates *S. aureus* (ear discharge), *P. mirabilis* (ear discharge), *P.aeruginosa* (wound), *S. pyogenes* (ear), *K.pneumoniae* (wound) *E. coli* (wound) and Coagulase negative staphylococci(wound) for the experimental study. Control strains [American Type Culture Collection *E. coil* ATCC 25922, *S. aureus* ATCC 25923, *P. aeruginosa* ATCC 27813, *K. pneumoniae*, *P. mirabilis* obtained from Ethiopia Health and Nutrition Research Institute (EHNRI)] were used as a quality control.

### Identification of multidrug bacterial isolates

Antibiotic susceptibility pattern of the isolated bacteria was done to identify multidrug resistant ones for the experimental study on Muller-Hinton agar (Oxoid, England) using disk diffusion method. Tetracycline (30 μg), penicillin G (10 μg), erythromycin (15 μg), gentamicin (10 μg), ciprofloxacin (5 μg), norfloxacin (10 μg), trimethoprim–sulfamethoxazole (25 μg), nitrofurantoin (300 µg), doxycycline (30 µg), ceftriaxone (30 μg), ampicillin (10 μg) and amoxicillin clavulanic acid (10 μg) (all Oxoid, England) were used to test the resistance pattern. Multidrug resistance was defined as non susceptible to ≥1 agent in ≥3 antimicrobial categories as per the recommendation of Magiorakos et al. (2012).

### Honey collection and processing

Six honey samples (three red and three white) from three districts were collected from local markets in sterile screwed-cup container and kept in cool and dry place in the laboratory for processing. Honey samples were first filtered with a sterile mesh to remove the debris and were stored at 2–8 °C for further use.

### Preparation of bacterial isolates

From the multidrug resistant colonies, three to five pure colonies were picked from each isolates with an inoculating wire loop, suspended in 4–5 ml of nutrient broth and incubated at 37 °C for 24 h. The bacteria suspension then was diluted with sterile distilled water until it matches the turbidity of 0.5 Mc Far land Standards (10^5^–10^6^ CFU/ml). The resulting suspensions were further diluted 1:100 in sterile nutrient broth to set inoculums density of 1 × 10^4^  CFU/ml according the set procedure (Kacaniova et al. 2011).

### MIC determination

The minimum inhibitory concentration of the honeys was determined using broth tube dilution method according to Kacaniova et al. (2011) procedure. Briefly, ten sterile test tubes were placed in rack, labeled each 1 through 8. Honey control tube (HC) and growth control tube (GC) were used as a quality control. One ml of freshly prepared nutrient broth was added to each tube, sterilized and cooled. Then one ml of undiluted honey solution 100 % was added to test tube number 1 and HC with a sterile micropipette and tips. Then twofold serial dilution was performed by transferring 1 ml undiluted honey into the second tube with separate sterile micropipette and tips and vortexed for homogenization. After a through mixing, 1 ml was transferred with another sterile micropipette from tube 2 and tube 3. These procedures continued until eighth tube with a dilution of 1:128 was reached and finally 1 ml was taken and discarded from tube 8. The GC tube received no honey was served as a growth control while the HC tube received no bacterial inoculums was served as a honey control.

Except the HC tube, each tube was inoculated with 1 ml of the culture of respective prepared organism. The whole procedures were repeated for all the organisms tested to each of the honeys. Tubes were then incubated at 37 °C for 24 h and observed by visual inspections for the presence and absence of growth (turbidity).

### MBC determination

To determine the MBC, incubated tubes showing no visible sign of growth/turbidity in MIC, were sub-cultured onto sterile nutrient agar plates by streak plate method and incubated at 37 °C for 24 h aerobically. The least concentration of honey that did not show growth of test organisms was considered as the MBC (Kacaniova et al. 2011). Then inoculated plates were scored as bactericidal if no growth; bacteriostatic if there is light to moderate growth and no antibacterial activity if there is heavy growth according the record of Payveld (1986).

### Pre test and data quality control

Pretest was conducted to check the method with quality control organisms. Sensitivity test was done against honey of different concentration and bacteria for its reliability and validity before it was used for actual experiment.

## Ethical consideration

The study was approved and ethically cleared by the Research and Ethical Review Committee of Mekelle University, College of Health Sciences (Ref. No: ERC 0459/2015). Written informed consent was obtained from each participant. Result of the bacterial and antimicrobial resistance profile of the bacteria were communicated with doctors in the ENT clinic and surgical wards during the study to help the patients.

## Results

The bacteriostatic activity of red honey from Atsbi area against *P. aeruginosa*, CoNS, *E. coli* and *K. pneumoniae* was 25 % (v/v), whereas white honey from the same area showed bacteriostatic activity against CoNS and *P. aeruginosa* at (50 % v/v), *K. pneumoniae* and *E. coli* at (25 % v/v), and *S. aureus* and *P. mirabilis* at *(*12.5 % v/v). The MIC of red honey from Temben area showed bacteriostatic activity at 6.25 % v/v for *S. aureus* and *S. pyogenes*, 12.5 % v/v for *P. mirabilis*, CoNS and *E. coli*, and 25 % for *K. pneumoniae* and *P. aeruginosa.* Mean MIC of 50 % was seen by the white honey of Temben for *K. pneumoniae*, *P. aeroginosa* and CoNS. Red and white honeys from Samre area were bacteriostatic at 12.5 % v/v for *E. coil*, CoNS and *S. aureus*, and 25 % v/v for *K. pneumoniae*. Over all, the mean MIC of all honeys for all tested multidrug resistant bacteria in this study was from 12.6 to 27.7 % v/v (Table 1).

**Table 1:** MIC% (v/v) of various honey samples against multidrug resistant bacterial isolates in Ayder Referral Hospital, January–May 2015

Honey dilutions										
Test bacteria	Net (1)	1/2	1/4	1/8	1/16	1/32	1/64	1/128	Areas	MIC% (v/v)
*S. aureus*	−	−	−	−	−	+	+	+	TR	6.25
*P. mirabilis*	−	−	−	−	+	+	+	+	TR	12.5
*P. aeruginosa*	−	−	−	+	+	+	+	+	TR	25
*CoNS*	−	−	−	−	+	+	+	+	TR	12.5
*S. pyogenes*	−	−	−	−	−	+	+	+	TR	6.25
*K. pneumoniae*	−	−	−	+	+	+	+	+	TR	25
*E. coli*	−	−	−	−	+	+	+	+	TR	12.5
*S. aureus*	−	−	−	−	−	+	+	+	TW	6.25
*P. mirabilis*	−	−	−	_	+	+	+	+	TW	12.5
*P. aeruginosa*	−	−	+	+	+	+	+	+	TW	50
*CoNS*	−	−	+	+	+	+	+	+	TW	50
*S. pyogenes*	−	−	−	−	−	+	+	+	TW	6.25
*K. pneumoniae*	−	−	+	+	+	+	+	+	TW	50
*E. coli*	−	−	−	−	+	+	+	+	TW	12.5
*S. aureus*	−	−	−	−	+	+	+	+	SR	12.5
*P. mirabilis*	−	−	−	−	−	+	+	+	SR	6.25
*P. aeruginosa*	−	−	−	−	+	+	+	+	SR	12.5
*CoNS*	−	−	−	−	+	+	+	+	SR	12.5
*S. pyogenes*	−	−	−	−	−	+	+	+	SR	6.25
*K. pneumoniae*	−	−	−	+	+	+	+	+	SR	25
*E. coli*	−	−	−	−	+	+	+	+	SR	12.5
*S. aureus*	−	−	−	−	+	+	+	+	SW	12.5
*P. mirabilis*	−	−	−	−	+	+	+	+	SW	12.5
*P. aeruginosa*	−	−	−	+	+	+	+	+	SW	25
*CoNS*	−	−	−	−	+	+	+	+	SW	12.5
*S. pyogenes*	−	−	−	−	+	+	+	+	SW	12.5
*K. pneumoniae*	−	−	−	+	+	+	+	+	SW	25
*E. coli*	−	−	−	−	+	+	+	+	SW	12.5
*S. aureus*	−	−	−	−	+	+	+	+	AR	12.5
*P. mirabilis*	−	−	−	−	−	+	+	+	AR	6.25
*P. aeruginosa*	−	−	−	+	+	+	+	+	AR	25
*CoNS*	−	−	−	+	+	+	+	+	AR	25
*S. pyogenes*	−	−	−	−	−	+	+	+	AR	6.25
*K. pneumoniae*	−	−	−	+	+	+	+	+	AR	25
*E. coli*	−	−	−	+	+	+	+	+	AR	25
*S. aureus*	−	−	−	−	+	+	+	+	AW	12.5
*P. mirabilis*	−	−	−	−	+	+	+	+	AW	12.5
*P. aeruginosa*	−	−	+	+	+	+	+	+	AW	50
*CoNS*	−	−	+	+	+	+	+	+	AW	50
*S. pyogenes*	−	−	−	−	−	+	+	+	AW	6.25
*K. pneumoniae*	−	−	−	+	+	+	+	+	AW	25
*E. coli*	−	−	+	+	+	+	+	+	AW	25

NB. *TR and TW* Temben red and white, *SR and SW* Samre red and white, and *AR and AW* Atsbi red and white

The bactericidal activity of red honey from Atsbi area against *P. aeruginosa*, CoNS, *E. coli* and *K. pneumoniae* was (50 % v/v); whereas the MBC of white honey from the same area showed bactericidal activity at (100 % v/v) for CoNS, *K. pneumoniae* and *P. aeruginosa*. Both red and white honeys from Temben area were bactericidal for CoNS, *K. pneumoniae* and *P. aeruginosa* at (100 % v/v), and red and white honey from Temben killed *S. aureus* at (50 % v/v) and (100 % v/v) respectively. MBC of both red and white honeys form Samre area killed *E. coli*, *S. aureus*, *CoNS* and *P. aeruginosa* at (50 % v/v). Over all the mean MBC of all honeys of tested multidrug resistant bacteria was from 30.4 to 75 % v/v (Table 2).

**Table 2:** MBC% (v/v) of honey against multidrug resistant bacteria in Ayder Referral Hospital, January–May 2015

Honey dilutions							
Test bacteria	Net (1)	1/2	1/4	1/8	1/16	Areas	MBC
*S. aureus*	−	−	+	++	+++	TR	50
*P. mirabilis*	−	−	−	−	++	TR	12.5
*P. aeruginosa*	−	+	++	+++	+++	TR	100
*CoNS*	−	+	++	+++	+++	TR	100
*S. pyogenes*	−	−	+	++	+++	TR	25
*K. pneumoniae*	−	+	++	+++	+++	TR	100
*E. coli*	−	−	+	++	+++	TR	50
*S. aureus*	−	−	+	++	+++	TW	50
*P. mirabilis*	−	−	−	++	+++	TW	25
*P. aeruginosa*	−	+	++	+++	+++	TW	100
*CoNS*	−	+	++	++	+++	TW	100
*S. pyogenes*	−	−	−	++	+++	TW	25
*K. pneumoniae*	−	+	++	+++	+++	TW	100
*E. coli*	−	−	+	++	++	TW	50
*S. aureus*	−	−	+	++	++	SR	50
*P. mirabilis*	−	−	−	++	+++	SR	25
*P. aeruginosa*	−	−	+	++	+++	SR	50
*CoNS*	−	−	+	++	+++	SR	50
*S. pyogenes*	−	−	−	+	++	SR	25
*K. pneumoniae*	−	−	+	++	+++	SR	50
*E. coli*	−	−	+	++	+++	SR	50
*S. aureus*	−	−	+	++	+++	SW	50
*P. mirabilis*	−	−	+	++	+++	SW	50
*P. aeruginosa*	−	−	+	++	+++	SW	50
*CoNS*	−	−	+	++	+++	SW	50
*S. pyogenes*	−	−	+	++	+++	SW	50
*K. pneumoniae*	−	+	++	+++	+++	SW	100
*E. coli*	−	−	+	++	+++	SW	50
*S. aureus*	−	−	−	+	++	AR	25
*P. mirabilis*	−	−	−	−	++	AR	12.5
*P. aeruginosa*	−	−	+	++	+++	AR	50
*CoNS*	−	−	+	++	+++	AR	50
*S. pyogenes*	−	−	−	−	++	AR	12.5
*K. pneumoniae*	−	−	+	++	+++	AR	50
*E. coli*	−	−	+	++	+++	AR	50
*S. aureus*	−	−	+	++	+++	AW	50
*P. mirabilis*	−	−	−	−	++	AW	12.5
*P. aeruginosa*	−	+	++	+++	+++	AW	100
*CoNS*	−	+	++	+++	+++	AW	100
*S. pyogenes*	−	−	+	++	+++	AW	25
*K. pneumoniae*	−	+	++	+++	+++	AW	100
*E. coli*	−	−	++	+++	+++	AW	50

−, no growth (bactericidal); +, light growth, ++, moderate growth (bacteriostatic); +++, heavy growth (no antibacterial potential)

The bacteriostatic activity of red and white honeys from Temben area for most control bacteria was from 6.25 to 12.5 % v/v. However, the MIC for *K. pneumoniae* (25 % v/v) was relatively higher than the other bacteria. All control bacteria except *P. aeruginosa* (25 % v/v), the bacteriostatic activity for honeys from Atsibi and Samre district was in the range of 6.25–12.5 % v/v. The concentration of Temben honey that killed the control bacteria was from 25 to 100 % v/v. Whereas honeys from Atsbi and Samre areas was from 25 to 50 % v/v. Red honey from Samre and Atsbi areas showed better antibacterial activity at lower concentration than the white honeys from the same areas. *P. aeruginosa* was completely killed at a higher concentration, 100 % v/v and relatively showed more resistant than the other control bacteria for all honeys in this study. In general the mean MIC and MBC of the tested honey against the control bacteria was from 7.7–12.6 to 25–55 % v/v respectively (Table 3).

**Table 3:** The mean MIC and MBC of various honey samples against control bacterial isolates in Ayder Referral Hospital, January–May 2015 (% (v/v))

Bacteria	Sites of honey collection											
	Temben area honey				Atsbi area honey				Samre area honey			
	RMIC	RMBC	WMIC	WMBC	RMIC	RMBC	WMIC	WMBC	RMIC	RMBC	WMIC	WMBC
*S. aureus*	6.25	50	12.5	50	6.25	25	12.5	50	12.5	12.5	6.25	50
*E. coli*	6.25	50	12.5	50	6.25	50	6.25	50	6.25	25	6.25	25
*P. aeruginosa*	12.5	50	12.5	100	12.5	25	25	50	12.5	50	25	50
*K. pneumoniae*	12.5	25	25	25	12.5	25	12.5	25	6.25	25	12.5	25
*P. mirabilis*	6.25	25	12.5	50	6.25	25	6.25	25	6.25	12.5	6.25	50
Mean	8.9	40	12.5	55	8.9	30	12.2	40	7.7	25	12.6	40

*RMIC* Red MIC, *WMIC* White MIC

## Discussion

The MIC and MBC value in this study indicated that all tested honeys have potential bactericidal and bacteriostatic activities against both control and test multidrug bacteria. This was similar to other studies conducted elsewhere (Allen et al. 2000; Getaneh et al. 2013; Ahmed et al. 2014). The finding of our current study indicated that *S. pyogenes* and *S. aureus* were completely inhibited at low concentration of honeys. This result was supported by the study finding of Kingsley who conducted study on the use of honey in the treatment of infected wound (Kingsley 2001).

The percentage by volume of honeys to completely prevent growth of *S. aureus*, *S. pyogenes*, *E. coli* and *P. mirabilis* was in the range of 6.25–12.5 % v/v, and for *P. aeruginosa* 12.5–50 % v/v. In contrary to this, a study conducted in Ethiopia has shown that the percentage by volume of honey to completely prevent growth of *E. coli*, *S. aureus* and *P. mirabilis* to be 6.5 % v/v and for *P. aeruginosa* 7.5 % v/v (Ahmed et al. 2014) which is lower concentration than our result. Another study by Willix has also found that the % (v/v) of Manuka honey to completely prevent growth for *S. aureus*, *S. pyogenes*, *E. coli*, *P. mirabilis* and *P. aeruginosa* was 1.8, 3.6, 6.0, 6.3 and 10.8 respectively (Willix et al. 1992). This difference in the antibacterial activity of honeys over place might be due to the difference in the species of bees (Mogessie 1994) and the differences in the test methods used and test organisms, where in our case we used multidrug resistant bacteria.

Study done in Ireland on Tazma honey have found 12.5 % v/v of MBC for Methicillin resistant *S. aureus* (MRSA), *E. coli* and *P. aeruginosa* (Cooper et al. 1999), and studies done from Ethiopia (Getaneh et al. 2013; Mogessie 1994) on the antibacterial activity of Tazma honey on MRSA bacteria have shown from 10 to 11.5 % v/v. This supports the idea that there is variation in the antimicrobial activities of honey by the source of flower and type of honey.

In our study red honeys from all sites showed better antibacterial activity at lower MIC and MBC for both control and test bacteria than the white honey, and this was in tandem to the study by Getaneh from Ethiopia (Getaneh et al. 2013). This was inline with the idea that color and consistency of honey is affected by the source of flower also by variables such as weather and climatic changes (Cooper et al. 1999). The difference of anti bacterial activity of honeys by color could also due to the difference in phenolic content of the honeys, which has strongly relation with its antioxidant activity of bacteria (Kek et al. 2014; Bertoncelj et al. 2007).

Our study revealed that *S. aureus*, *S. pyogenes*, *E. coli* and *P. mirabilis*, were more sensitive at lower MIC and MBC of honey than the *P. aeruginosa*, *K. pneumoniae* and *CoNS*. This was supported by result of Getaneh et al. from Ethiopia who conducted research on the in vitro assessment of the antimicrobial effect of Ethiopian multi flora honey on MRSA (Getaneh et al. 2013). Possible reason for these variations between bacteria response to honeys might be due to difference in their cellular organization of the bacteria and the variation in honeys. The bactericidal activity of the honeys for all tested isolates in this study was found to be between 50 and 100 % v/v concentration. This was comparable to other researchers from other palaces (Kingsley 2001; Ahmed et al. 2014). The bactericidal concentrations of honey against *S. aureus* in our study was between 50 and 100 %. This concentration was higher than the finding of other researchers (Molan 1992; Ahmed et al. 2014; Molan and Betts 2000).

*Pseudomonas aeruginosa* was reported to be resistant to honey by Efem (1988); in contrary to this result however, the bacteria was sensitive to all honeys tested in this study, though at it was a bit at higher concentration than the other bacteria. Our result was also supported by the study done in other part of Ethiopia (Ahmed et al. 2014). The high resistance *P. aeruginosa* to honeys could be due to the low permeability of its cell wall, genetic capacity to express resistant mechanisms and mutation in chromosomal genes which regulate resistance genes (Allen et al. 2000).

Both red and white honeys from all areas in this study have shown antibacterial activity against *K. pneumoniae.* This was in line with the report by other researchers (Allen et al. 2000; Anyanwu 2011; Subrahmanyam 1991). Our result was however, in contrast with studies by Ahmedet et al. from Ethiopia (Ahmed et al. 2014) and Olawuyi et al. (2010) who studied on antibacterial activities of honey from different location and reported no bactericidal activity against *K. pneumoniae.* The variations in sensitivity could be attributed to differences in growth rate and lower cell wall permeability of pathogen, nutritional requirements, temperature, inoculums size and difference in honeys and the test method used (Molan and Betts 2000).

In general honeys tested from the three different areas showed varied bacteriostatic and bactericidal activities against the tested multidrug resistant bacteria. However, pharmacological standardization and clinical evaluation on the effect of honey are essential before using honey as a preventive and curative measure to common diseases related to the tested bacterial species. This bacteriostatic and bactericidal activity was different by sites of collection and colour of the honeys. Honey from Samre area showed relatively better bacteriostatic and antibacterial activity than Temben and Atsbi districts. This could be due to the variations in hydrogen peroxide, crops and vegetation’s in these districts available for honeybees to make honey. Both control and test *K. pneumoniae* and *P. aeruginosa* were inhibited at relatively higher concentration of honey than the other bacteria in this study. This might be related to nature of the bacteria i.e. low permeability of cell wall, genetic capacity to express resistant mechanisms and mutation in chromosomal genes which regulate resistance genes. Red honeys in this study showed better anti bacterial activity against both control and test bacteria than the white honeys.
